# The Information Value of Non-Genetic Inheritance in Plants and Animals

**DOI:** 10.1371/journal.pone.0116996

**Published:** 2015-01-20

**Authors:** Sinead English, Ido Pen, Nicholas Shea, Tobias Uller

**Affiliations:** 1 Edward Grey Institute, Department of Zoology, University of Oxford, Oxford, United Kingdom; 2 Theoretical Biology Group, Centre for Ecological and Evolutionary Studies, University of Groningen, Groningen, The Netherlands; 3 Department of Philosophy, King’s College London, London, United Kingdom; 4 Department of Biology, Lund University, Lund, Sweden; Blaustein Institutes for Desert Research, Ben-Gurion University of the Negev, ISRAEL

## Abstract

Parents influence the development of their offspring in many ways beyond the transmission of DNA. This includes transfer of epigenetic states, nutrients, antibodies and hormones, and behavioural interactions after birth. While the evolutionary consequences of such non-genetic inheritance are increasingly well understood, less is known about how inheritance mechanisms evolve. Here, we present a simple but versatile model to explore the adaptive evolution of non-genetic inheritance. Our model is based on a switch mechanism that produces alternative phenotypes in response to different inputs, including genes and non-genetic factors transmitted from parents and the environment experienced during development. This framework shows how genetic and non-genetic inheritance mechanisms and environmental conditions can act as cues by carrying correlational information about future selective conditions. Differential use of these cues is manifested as different degrees of genetic, parental or environmental morph determination. We use this framework to evaluate the conditions favouring non-genetic inheritance, as opposed to genetic determination of phenotype or within-generation plasticity, by applying it to two putative examples of adaptive non-genetic inheritance: maternal effects on seed germination in plants and transgenerational phase shift in desert locusts. Our simulation models show how the adaptive value of non-genetic inheritance depends on its mechanism, the pace of environmental change, and life history characteristics.

## Introduction

Parents contribute to offspring development in many ways, including transmission of DNA and its associated epigenetic marks, allocation of resources to the egg and growing embryo, and behavioural interactions following birth or hatching. The mechanisms by which parents affect offspring development in addition to DNA transfer are known as parental effects or non-genetic inheritance [1, 2]. The growing literature on the role of non-genetic inheritance in evolution is heterogeneous and has developed along several main themes [3]. A wide range of theoretical models in maternal effect, niche construction, and cultural inheritance research have shown that non-genetic inheritance can influence the course of evolution by affecting individual fitness, disconnecting what is selected from what is inherited, modifying selection on future generations, and affecting higher organizational levels, including the facilitation of genetic differentiation and speciation (reviews in [4–11]). While such consequences of non-genetic inheritance for phenotypic and genetic evolution are increasingly recognized, relatively little is known about the conditions under which non-genetic inheritance is itself adaptive [12, 13]. It has been proposed that non-genetic inheritance can enable co-adaptation of plastic responses in parents and offspring and hence matching offspring phenotype to local conditions [14–19]. For example, maternal determination of diapause and dispersal could increase offspring fitness by avoiding adverse environments [20], and social learning from parents may enable transfer of knowledge about what is safe to eat, how to find food, and how to avoid predators [21–24]. However, most of these suggestions rely on verbal arguments, with few explicit models of how non-genetic inheritance evolves, in particular outside of studies of cultural evolution (e.g., [25–30]). There has also been little attention paid to the different mechanisms of non-genetic inheritance (e.g. active transmission of cues by parents versus incomplete resetting of epigenetic marks, [31]), both in terms of when they would be selected and their consequences for evolution (for the latter see [32–34]).

It is the adaptive value of non-genetic inheritance that we address in this paper. We do so by presenting a framework based on the assumption that, from the perspective of a developing organism, both genetic and non-genetic inputs can be potential sources of information about the selective regime facing an individual [35, 36]. Evolution of phenotype determination, such as genetic versus environmental morph determination, can therefore be expressed in terms of responsiveness to those cues. Here, we show that this framework has the attractive feature that adaptive evolution of phenotype can be expressed in terms of mutual information, which quantifies the extent to which observation of one random variable reduces uncertainty about another random variable ([37]; see S1 File). We subsequently make use of this framework to generate two individual-based simulation models and derive conditions that select for the commonly observed maternal effects on diapause in plants [38] and transgenerational phase shift in locusts [39]. These models serve to exemplify the key aspects of environmental heterogeneity and endogenous mechanisms that favour the evolution of non-genetic inheritance of phenotypes.

### Conceptual Framework

**Figure 1:**
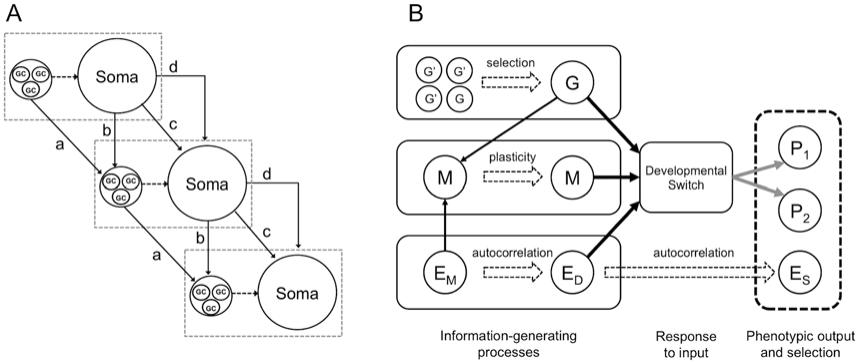
Inheritance pathways and model framework. A. Different pathways of inheritance, including (a) direct germline transmission between germ cells (GC) across generations (e.g inheritance of DNA or DNA methylation marks due to incomplete resetting); (b) effect of parental phenotype on germ cells of next generation (e.g. environmentally-induced changes in methylation status in early embryos); (c) effect of parental phenotype on offspring phenotype (e.g. large mothers producing large offspring); (d) effects of parental phenotype on offspring environment (dotted box) (modified in part from [11]). B. The model framework: evolution of a developmental switch that can respond to three sources of input, genetic loci (G), maternal phenotype (M), and environment (E). All three sources of input can carry information as follows (left hand side, from top to bottom). First, at the outset, G arises by a random process (e.g. mutation from G’ ◊ G) and leads to, and correlates with, phenotype P_*i*_; after one or several rounds of selection, G correlates both with P_*i*_ and with some environmental factor(s) experienced by the offspring E_S_ in virtue of which G was selected. Second, the maternal phenotype can carry correlational information about the selective regime in E_S_ if it correlates with G and/or if the maternal environment that influences the expression of M (E_M_) shows autocorrelation with the environment of the offspring at the time of selection (E_S_). Finally, the environment of development (E_D_) can carry correlational information about the environment of selection (E_S_). Note that figure B omits additional links arising from, for example, niche construction, where ancestors affect E_s_ directly [5] and genetic differences that cause variation in parental contribution to offspring (e.g. [83]). All three sources of input can affect the liability of a developmental switch (thick black arrows), which in response can produce a range of phenotypes (grey arrows), *P_i_* (here *i* = 2). Phenotypes are subject to selection (dashed box) as a result of variation in the environment (*E_S_*). Thus, adaptive evolution of the developmental switch is expected to increase the match between phenotype, *P_i_*, and the selective environment, *E_S_*, by modifying the response to genetic, maternal, or environmental input.

Our framework builds on the work of Leimar and colleagues [35, 40]. They showed how both genetic and environmental inputs can be seen as cues about coming selective regimes, and that differential use of these cues by a developmental switch can be understood as different degrees of genetic and environmental morph determination (see also [41]). More recently, the same approach has been used to propose a distinction between inheritance systems based upon how inheritance mechanisms come to carry information [36]. Here we expand this perspective to encompass all forms of non-genetic inheritance in the broad sense of any causes of offspring development, in addition to DNA, that are contributed by parents (Fig. 1A). This includes many causes that are commonly referred to as parental effects [1, 42]. It also includes stable, phenotype-independent, transmission of epigenetic states, such as DNA methylation (the degree to which states are passed on unchanged we call ‘transmission stability’) [31, 43, 44]. Furthermore, non-genetic inheritance can also involve direct effects of the environment if the state of the environment is directly affected by the parent (Odling-Smee [45] and Jablonka & Lamb [2] provide illustrative examples of these mechanisms).

The general framework for addressing when these different forms of non-genetic inheritance will be favoured by selection is outlined in Fig. 1B. We consider phenotypic evolution from the perspective of a developmental switch, i.e., a mechanism by which organisms can develop alternative phenotypes and that itself is reliably inherited between generations. This switch can respond to different sources of inputs: allelic variation at genetic loci, the maternal phenotype or its products, and the external environment (Fig. 1B). Allelic variation represents the genetic inheritance system, the maternal phenotype different forms of non-genetic inheritance mechanisms, and the external environment either some form of ecological inheritance (if there is a causal link from the parent or earlier ancestors to the environment [45], such as territory acquisition) or simply the environment that is not affected by parents (which may vary in resource availability or predation risk, for example).

The developmental switch can produce a range of different phenotypic outcomes, but for simplicity we consider only two possible phenotypes (see details below). We refer to these phenotypes as morphs. Evolution of morph determination thus occurs when the developmental switch changes its responsiveness to different sources of input, for example, a transition from responding primarily to allelic variation at one or several genetic loci to responding primarily to environmental variation. Evolution may also favour responsiveness to several types of cues. We have shown previously that such transitions can be captured in informational terms [36]. This is because both genetic and non-genetic inputs can carry information about the future selective regime which, here, is a function of the external environment encountered as an offspring. First, if the environment at the time of development of the morph (*E*_D_) correlates with the environment at the time of natural selection on the morph (*E*_S_), then *E*_D_ carries correlational information about *E*_S_. Similarly, the parental phenotype can carry correlational information about *E*_S_, for example, if the parental phenotype responds to its own environment and the parental environment correlates with offspring environment. Finally, because natural selection will tend to build up gene frequency differences between environments, genotype can also correlate with *E*_S_ if different genotypes have different fitness across environments and there is incomplete admixture in between rounds of selection (e.g., due to limited dispersal between environments, [35]).

Under this information framework, there are several reasons why individuals might rely on certain types of information or not. Signals can be noisy, for example if the cues provided by the parental phenotype are obscured by other individuals or the external environment. Individuals may also incur costs in sensing or processing the signal. Finally, even when a reliable signal is available, there may be costs and benefits of using it, including costs of detection, or of false positive or false negative responses.

Although all three sources of input can carry correlational information about the environment at the time of selection, the information carried by the genotype differs from that of the external environment and the parental phenotype in one important respect: the build-up of information in genotype is the result of natural selection on stably transmitted (or replicated) variants ([35, 36, 40] Fig. 1B). This means that when environmental cues are unreliable, dispersal between environments is limited, and selection is sufficiently strong, differences in allele frequencies between environments will build up and the system will evolve towards genotypic morph determination [35, 46, 47]. In contrast, environmental or parental morph determination should evolve when genetic differences between environments cannot be sustained due to gene flow, and when the parental phenotype or the offspring environment provides reliable cues to the selective environment.

This model approach presents a powerful way to evaluate the life-history and environmental conditions that favour non-genetic inheritance as opposed to genetic determination of phenotype or within-generation phenotypic plasticity. To exemplify the use of this approach for understanding the evolution of non-genetic inheritance we build individual-based simulation models. Specifically, we model two frequently discussed examples of adaptive non-genetic inheritance: maternal effects on the timing of germination in plants [38] and transgenerational plasticity of morph development in locusts [48]. These examples highlight the basic logic of the framework and enable us to make a number of specific and more general predictions about the conditions that favour non-genetic inheritance through some of the pathways exemplified in Fig. 1. We also use a simple, analytical version of this model to show that natural selection will favour switches that respond to different sources of inputs such that the mutual information between phenotype and the environment at the time of selection is maximized (S1 File).

### Simulation Models

In our first simulation model, we consider a plant-like system with limited dispersal of seed and pollen between patches, and where the trait under consideration is seed germination (which can be influenced by the light environment of the parent, as indicated by their phenotype). In this system, we consider the evolution of a particular type of non-genetic inheritance: that of offspring responses to variation in parental phenotype. We investigate the effects of spatial environmental heterogeneity and sex-specific gamete dispersal on the evolution of this non-genetic inheritance. There are several routes by which parents can influence their young, however, with potentially contrasting consequences for adaptive evolution (review in [9]). Our second simulation model is based on phase change in desert locusts, which involves two pathways of maternal inheritance: a direct effect of an environmentally responsive maternal phenotype and (partial) inheritance of this effect down generations independently of the local environment (Fig. 1). This captures one aspect of recently discussed ‘semi-stable’ inheritance of epigenetic states, such as DNA methylation, where epigenetic variants can be transmitted down several generations in the absence of the inducing environment ([31, 43]; see below). This system also allows us to address the role of temporal, rather than spatial, variation in the environment for the evolution of non-genetic inheritance.

We first describe the shared features of the two models followed by details for how the framework is adapted to seed germination in plants and phase shift in locusts. In both cases we consider a population of individuals with two possible phenotypes, *P*_1_ and *P*_2_, in an environment that can take on one of two different states, *E*_1_ and *E*_2_. Phenotype *P*_1_ has higher fitness than phenotype *P*_2_ in *E*_1_, and vice versa for *E*_2_. Mortality of mismatched phenotypes is equal to *s* and relative fitness is thus defined as 1-*s*. For simplicity we consider non-overlapping generations in both of our models. The developmental regulation of morph expression is determined by the action of a developmental switch that can respond to three types of input: allelic variants on one or more genetic loci, stably transmitted from parents to offspring (genetic inheritance), the parental phenotype in some form that can causally affect offspring development (non-genetic inheritance), and the offspring’s external environment at the time of morph determination (Fig. 1B). Non-genetic inheritance could be, for example, maternal body size or some form of parental behaviour that may influence offspring development. It could also be an epigenetic mark, such as the level of methylation of a particular DNA sequence in the parental zygote. In principle, the parental phenotype can be affected by genetic, direct environmental and parental effects, and the environment can be modified by the offspring or the parental phenotype. However, in our examples below, most of these interactions are not part of the simplified biology of the system.

The three inputs combine into a single liability, *y*, which, if exceeding a fixed threshold, *T*, results in development of phenotype *P*_1_. If *y* does not exceed the threshold, the switch develops phenotype *P*_2_. Thus, the liability of morph determination in offspring environment *E_S_* ∈{*E*_1_, *E*_2_} can be written

$$y=y_{G}+b_{M}f(E_{M},P_{M})+b_{F}f(E_{F},P_{F})+b_{O}E_{O}+\varepsilon$$ [1]

in which *y*_G_ is the effect ascribed to inherited genes; *b*_M_, *b*_F_ and *b*_O_ are the relative weights given to maternal, paternal and environmental cues respectively; *f(E*_i_ *, P*_i_) is a function describing how the parental cue is affected by parental environment and phenotype (maternal or paternal, depending on whether *i* is *M* or *F*, respectively); *E*_O_ describes the offspring’s assessment of its own environment; and is *ε* an error term (developmental noise). In general, the weighting terms *b*_M_ and *b*_O_ are continuous variables that can take on both positive and negative values and parameters describing parent and offspring phenotype or environment (*P*_i_, *E*_i_ and *E*_O_) can be discrete variables or continuous variables depending on the system.

From this general model we can subsequently specify the different functions, number of genetic loci, type and extent of environmental heterogeneity, and life history of the organism (e.g., dispersal probability, mode of reproduction). This enables specific models that test the conditions that favour evolution of non-genetic inheritance of morphs, relative to genetic inheritance and within-generation plasticity, which we here exemplify with two case studies. In both case studies, we verified that the variables reached a stationary state in our simulations and present results from ten replication simulations for each setting.

### Model 1: Maternal effects on seed germination

In many plant species, the parental environment has a strong effect on traits expressed early in life in the next generation [49, 50]. One of the best studied of such traits is the timing of germination, which also influences subsequent life history [51]. Several authors have suggested that maternal effects on germination are an adaptation to the largely sedentary life of plants, which means that they are subject to a patchy environment at the level of the population, but consistent conditions within lineages (or families) across generations [38, 49, 52–54]. For example, in the monocarpic herb *Campanulastrum americanum*, seeds from mothers in light conditions tend to germinate in the autumn and those in shade conditions in the spring [38], although there is also a direct environmental effect on the timing of seed germination. Galloway and Etterson [38] used a factorial design to show that the timing of germination of seeds results in higher fitness when the maternal and offspring environments are matched. This example is potentially an adaptive maternal phenotypic effect [49], whereby parental phenotypic adjustment to the environment (e.g. growing to a taller height in light gap) causes physical or physiological changes in the seed that in turn influences their timing of germination (these types of effects are sometimes referred to as anticipatory maternal effects [55], adaptive transgenerational plasticity [54], and detection-based non-genetic inheritance [36]).

In these plant systems, with limited dispersal between patches, it seems plausible that parents should play a role in adaptive morph determination of their offspring. There are several open questions as to the relative importance of non-genetic inheritance compared to genetic inheritance and within-generation plasticity. A recent study on the *Claytonia perfolata* polyploid complex demonstrated that the impact of adaptive transgenerational effects on leaf morphology in response to shade environment was small relative to within-generation plasticity and local adaptation [56]. As discussed above, the information content of the parental phenotype determines the extent to which offspring come to rely on parents for adaptive morph determination, and this can be affected by environmental and life history characteristics of particular systems, such as the extent of dispersal between environments and the frequency with which one environment is likely to be encountered. In principle, both maternal and paternal effects are possible, but maternal effects are usually considered to be stronger because mothers have more opportunity to influence their offspring’s early environment. There is growing evidence that fathers also play a role, both in modification of epigenetic marks [57] and later environmental effects [58]. Another possibility for greater emphasis on maternal input is that sex-biased dispersal in gametes can affect the reliability of information received from fathers versus mothers. Indeed, a recent model has shown that sex-biased dispersal can result in the emergence of uniparental inheritance of traits [25].

**Figure 2:**
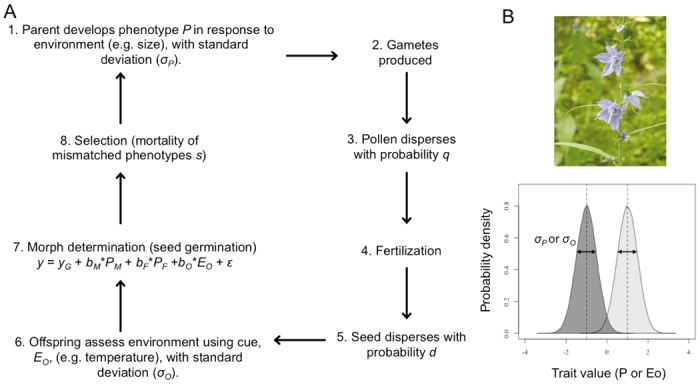
Plant model overview. A. Life history of plant model as explained in detail for each step, with; B. Upper: picture of *C. americanum*, the perennial herb species which provides the biological motivation for this model; lower: a schematic explaining the role of the variable parameters *σ*_P_ and *σ*_O_ in determining the environment-specificity of the parental phenotype or offspring cue, both assumed to be Gaussian traits with standardized means of-1 and 1 in the shade and light environments respectively. Image: L. Galloway.

Here, we formalize verbal suggestions regarding the adaptive significance of maternal effects on seed germination using a life history characterized by that of *C. americanum*, as outlined in Fig. 2. In our model, organisms are diploid, obligatorily outcrossing hermaphrodites (i.e., with sexual reproduction). The environment consists of randomly distributed patches with two distinct types (shaded versus light gap), and the offspring phenotype is equivalent to spring or autumn germination, which is favoured in shade or light patches, respectively. Liability for autumn or spring germination can be influenced by genetic loci and how offspring weigh their parents’ phenotype and their own assessment of the environment, i.e.:

$$y=y_{G}+b_{M}P_{M}+b_{F}P_{F}+b_{O}E_{O}+\varepsilon$$ [2]

where *y*_G_ is the effect ascribed to inherited genes; *b*_M_, *b*_F_ and *b*_O_ are the relative weights given to maternal, paternal and offspring inputs respectively; these inputs being maternal and paternal phenotype (*P*_M_ and *P*_F_ respectively) and offspring assessment of its own environment (*E*_O_); and *ε* is a developmental-noise error term.

We use this model to test the conditions under which adaptive maternal effects evolve, relative to genetic, paternal and direct environmental inputs. We vary the parameters of environment type frequency, extent of seed and pollen dispersal, environment-specific parental trait distribution and offspring assessment of the environment (Table A in S2 File). This enables us to investigate the conditions under which maternal phenotype (and maternal environment), paternal phenotype (and paternal environment), genetic and direct (offspring) environment inputs to morph determination evolve in this system. Below, we present an overview of the key model results, with full details provided in S2 File.

**Figure 3:**
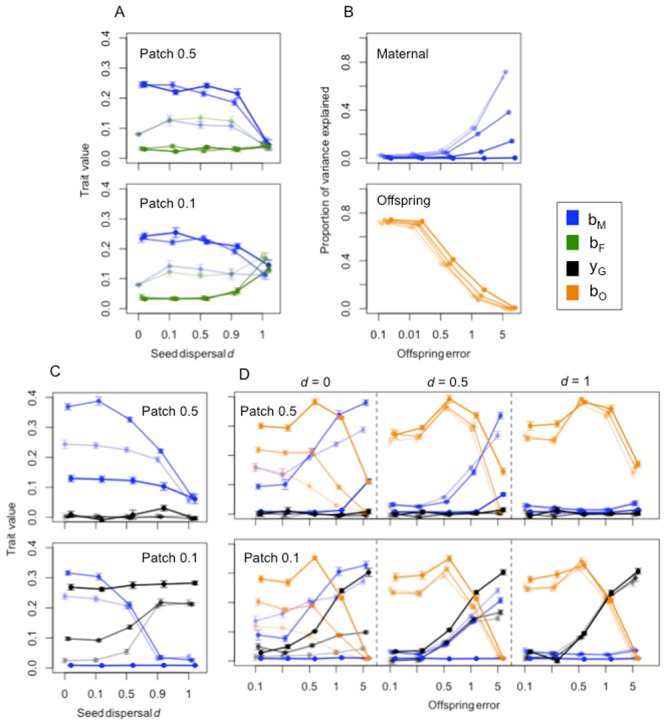
Plant model results. (A, C, D) Mean ± SE evolved values from the last 20,000 generations for each loci for simulations where environments are equally common (top) or one patch is rare (bottom); and (B) proportion of variance explained by maternal (top) or offspring (bottom) environment using analysis of variance of the trait liability *y* (as explained in S2 File). Blue points represent values for maternal input *b_M_* (A, C, D), green points, paternal input *b_F_* (A), orange points, offspring input *b_O_* (D) and black points, genetic input *y_G_* (B, D). Separate lines of increasing darkness represent increasing levels of pollen dispersal (A) or parental trait distribution (B-D). Values are provided in separate panels for three levels of seed dispersal in panels of plot D. The patterns in B are qualitatively similar if the evolved values are plotted (see Figure C in S2 File).

**Seed germination model: summary of results.** First, we consider the evolution of maternal versus paternal effects under varying levels of seed and pollen dispersal, depending on whether patches are equally distributed or one patch is relatively rare. As expected, given that there is no genetic or offspring input and the only information offspring have when determining their phenotype is through parental effects, we find that maternal effects evolve under most conditions (Fig. 3A) apart from when seed dispersal is high and patches are equally frequent (where random determination provide an equally high proportion of matching phenotypes). By contrast, paternal effects are generally selected against, unless pollen does not disperse (Fig. 3A). When one patch is rare and seed dispersal is high, maternal and paternal effects both evolve (Fig. 3A, bottom), with the result that the phenotype matching the most common environment is always produced.

Second, we investigate the relative influence of maternal effects and offspring plasticity under varying maternal trait and offspring environment-cue distributions, in the absence of genetic or paternal input. We find that, although seed dispersal is low, morph determination is largely based on direct offspring environment input rather than on maternal phenotype, unless the offspring environment cue has low information value (Fig. 3B, top) Maternal effects evolve when offspring environment cue shows low specificity, particularly when there is little overlap in maternal phenotype between environments (Fig. 3B, bottom).

Third, we consider maternal versus genetic morph determination depending on seed dispersal, patch frequency and parental trait distribution (i.e., there is no offspring plasticity or paternal effect). We find that, when patches are found in equal numbers, genetic determination does not evolve whereas maternal effects evolve (unless seed dispersal is very high), with greater values when the parental phenotype is narrowly distributed between environments (i.e., highly environment-specific; Fig. 3C, top). When one patch is rare, however, genetic input to morph determination is selected for, particularly if the parental phenotype overlaps widely across environments and, for other values of parental trait distribution, for high levels of seed dispersal (Fig. 3C, bottom).

Finally, we combine these insights to address the general conditions—of seed dispersal, parental trait distribution, offspring cue distribution and patch frequency— under which genetic, maternal and offspring environment input to morph determination evolve (assuming no paternal input and high pollen dispersal, consistent with our result that paternal effects are of limited importance in this system). We find that genetic determination only evolves when one patch is rare, when offspring cue is uninformative and (if seed dispersal is limited) when parent traits overlap across environments (Fig. 3D, bottom). Maternal effects evolve when offspring cue is uninformative and seed dispersal is low (Fig. 3D). Direct offspring input evolves under most scenarios, apart from when offspring cue is uninformative, and offspring are less sensitive to their own environment when seed dispersal is low and parent traits do not overlap across environments (Fig. 3D).

### Model 2: Transgenerational phase shift in locusts

Desert locusts (*Schistocerca gregaria*) exhibit remarkable phenotypic plasticity in response to crowding conditions, with solitary and gregarious morphs (termed ‘phases’) differing in a suite of morphological and behavioural traits [39, 59, 60]. Crowding occurs when population densities increase. This process is reinforced by the fact that once phase shift is initiated, individuals become attracted to rather than avoid conspecifics. An individual’s phase is also influenced by the conditions experienced by its mother, with a likely mechanism being a gregarizing agent added to the foam surrounding the eggs [61]. The exact mechanism underlying phase change within and across generations has yet to be elucidated, although there is a suggestion that DNA methylation may play a role [62]. The transition from a largely solitary population to a predominantly gregarious one occurs in fewer generations than the other way around, termed a hysteresis effect [61]. Such an effect suggests the presence of a ‘phenotypic memory’ that could indicate incomplete resetting of epigenetic states [31, 63, 64].

The adaptive significance of partial resetting of epigenetic marks should depend on the benefits in terms of maintaining the correct phenotype in the presence of incorrect environmental and maternal cues relative to the costs in terms of the number of generations it takes to switch from one phenotype to the other in the presence of a real environmental shift [9]. As such, errors in perceiving the environment, the cost of exhibiting a mismatched phenotype and the probability of the environment switching may all affect when transmission stability evolves. It has been suggested that the hysteresis effect in locusts might be adaptive because of differences between environments in the likelihood of detecting the wrong environment: it is possible for a gregarious individual to be separated from the crowd accidentally, yet highly unlikely that a solitary individual should find itself in a crowded situation erroneously [65]. Moreover, the cost of phenotypic mismatch may be higher for aposematic gregarious individuals in uncrowded environments than for cryptic solitary individuals in crowded environments ([65]). This verbal suggestion has yet to be tested in an explicit evolutionary model (but see [66] for a population dynamic approach).

**Figure 4:**
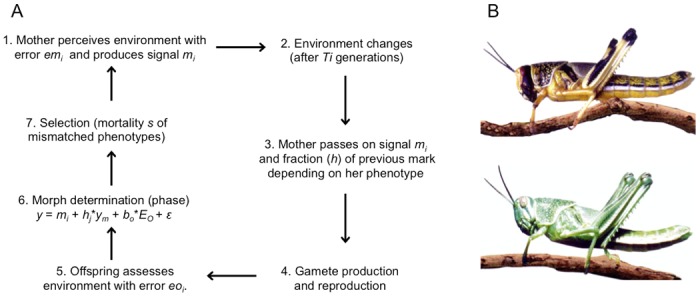
Locust model overview. A. Life history of locust model as explained in detail for each step, with a picture of the two phase states of the desert locust (solitary on top, gregarious on bottom); B. The non-genetic inheritance pathways relevant to this model, i.e. both a direct maternal effect depending on her environment (*m*), and a fraction of the previous generation’s parental mark transmitted through the germline (*h*). Image: C. Tucker.

Here, we use simulations to explore the conditions under which maternal effects on morph determination and a hysteresis effect evolve. The model is similar to the basic model described above, although the population is spatially homogenous, with no dispersal, and the environment fluctuates between crowded and uncrowded states. The offspring phenotype is solitary or gregarious, which have relatively higher fitness in the uncrowded and crowded environments respectively. The life cycle is explained in Fig. 4. The mother transmits a substance (e.g. gregarizing agent which determines expression of hormone influencing phase determination) to her offspring depending on the environment she perceives. The amount of maternal effect passed on to offspring is a sum of this direct maternal environment input and a fraction of the maternal input from the previous generation, i.e, an input not erased or reset at reproduction, which corresponds to transmission stability. Offspring can add to the inherited amount of maternal input depending on their own estimate of the environmental state. Thus, liability to develop a solitary or gregarious morph is:

$$y=m_{i}+h_{j}y_{m}+b_{o}E_{O}+\varepsilon$$ [3]

where *m*_i_ is the maternal environmental input (i = crowded or uncrowded), *h*_j_ is the transmission stability of the previous maternal input (i.e., *y*_m_ = *m*_i[t-1]_ *+ h*_j_ * m_i[t-2]_), depending on maternal phenotype (j = solitary or gregarious), *b*_o_ is weighting on offspring environment input (*E*_O_) and *ε* is a developmental-noise error term.

We use this model to investigate, first, how variation in maternal and offspring error in assessing the environment affects the evolution of different mechanisms of non-genetic inheritance (direct maternal effects versus transmission stability). We then consider the conditions under which hysteresis evolves, by allowing asymmetry between environments in selection on mismatched phenotypes, the number of generations at which the environment remains in a certain state, and in the probability mothers or offspring receive an incorrect signal about their environment (maternal or offspring error, respectively). Finally, we compare the conditions under which maternal effects evolve when offspring are also allowed to assess their own environment. We provide an overview of our key findings below, with full details in S2 File.

**Figure 5:**
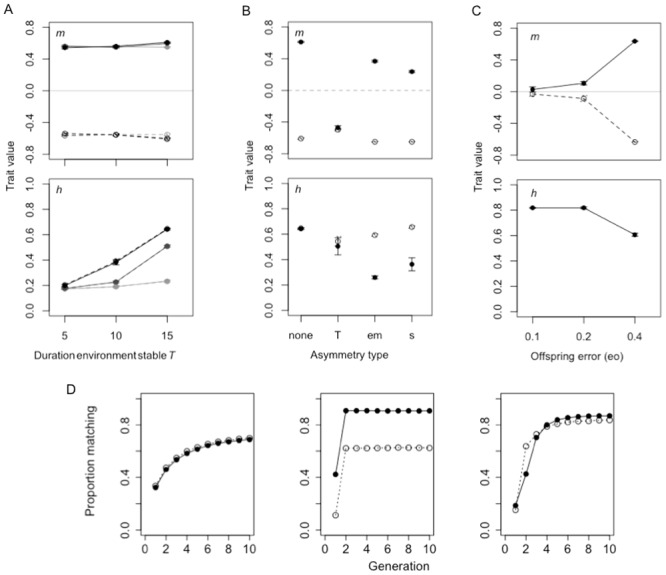
Locust model results. (A-C) Evolved values (mean±SE from last 20,000 generations) for, top panel, maternal environment effects *m* (filled circles and solid lines denote crowded environment, hollow circles and dotted lines denote uncrowded environment) and, bottom panel, phenotype-specific transmission stability *h* (filled circles and solid lines represent gregarious phenotype, hollow circles and dotted lines, solitary phenotype). (A) Separate values are shown for increasing levels of maternal error (pale grey = 0.1, dark grey = 0.2, black = 0.4). (B) Labels on x-axis explain asymmetries considered, i.e. T = (15,5); em = (0.4,0.1) and s = (0.2,0.1) for (uncrowded, crowded) environments respectively. (D) Example of how to measure the hysteresis effect: proportion of matched phenotypes in the population (from one simulation run) for 15 generations following an environmental switch (i.e. duration the environment is stable), when (left) there is no asymmetry in maternal error, i.e. em = (0.4,0.4), (centre) there is asymmetry in maternal error only (em = (0.4,0.1)) but transmission stability is fixed at zero and (right) there is both asymmetry in maternal error and transmission stability.

**Locust phase shift model: summary of results.** We first examine how environmental stability and maternal error affect the evolution of direct maternal environmental effects and transmission stability of the maternal input received in the previous generation, assuming no genetic or offspring input. We find that maternal environmental effects evolve under all conditions and transmission stability evolves when maternal error is high and environments are relatively stable (Fig. 5A). The degree of transmission stability depends on the period of environmental stability, the extent of environment-specificity of the maternal effect, and the strength of natural selection on offspring producing the phenotype appropriate to their environment. Accordingly, asymmetries in environmental stability, the probability of maternal error and selection result in differences in the evolved values of maternal environmental effects and phenotype-dependent transmission stability (Fig. 5B). However, a hysteresis effect— as indicated by asymmetries in how rapidly the proportion of individuals in a population produce a phenotype matched to a new environment following a switch in one direction or another— only occurs when maternal accuracy or selection varies between environments (Fig. 5D), but not if one of the environmental states lasts for longer than the other (Figure I in S2 File).

Finally, we investigate the evolution of non-genetic inheritance—both in forms of the direct maternal effect and transmission stability—when offspring have the opportunity to assess their environment directly, with varying degrees of accuracy. We assume the conditions favouring transmission stability when no offspring assessment occurs (i.e., long periods of environmental stability and mothers likely to receive the wrong cue about their environment; Fig. 5A). Under these conditions, offspring are selected to weigh their own environment to a greater extent than the cues transferred from the previous generation, and maternal environmental effects and transmission stability only evolve when the probability of offspring error is high (Fig. 5C). Hysteresis occurs when the probability of offspring error is high, and when the probability of maternal error differs between environments (Figure M in S2 File).

## Discussion

In this study, we apply an information-based approach to investigate the evolution of non-genetic inheritance mechanisms, using simulation models of two ecologically relevant scenarios: determination of germination strategies in plants, and of phase state in locusts. In spite of the contrast between the systems –spatial versus temporal environmental variation, different mechanisms of non-genetic inheritance —we find some robust results that are common to both systems. First, adaptive evolution of non-genetic inheritance mechanisms depends on the likelihood of encountering an environment that correlates with the environment or the phenotypes of parents in previous generations. This, in turn, is affected by the distribution of environments, dispersal and the dynamics of environmental change, which must occur at a scale at which non-genetic inheritance rather than genetic inheritance is favoured. Second, variation in information content of the maternal, genetic or offspring cue, i.e., the extent to which each channel carries correlational information regarding the selective regime experienced by offspring, results in varying degrees of genetic, parental or offspring morph determination. Systems that rely on more than one source of input are likely to be widespread. Nevertheless, both models find that, under most conditions, direct effects of the environment tend to predominate, unless offspring are particularly limited in their ability to extract or assess cues from their environment, or if the environment is highly variable or noisy. The models also produce some system-specific insights of interest, such as the effect of sex-specific gamete dispersal on the evolution of paternal versus maternal effects (plant model) and how different mechanisms of non-genetic inheritance can contribute to different transgenerational transition rates from one morph to the other (locust model). Below, we discuss these findings in more detail and suggest some additional uses of the framework for studying more complex scenarios.

Both genetic and non-genetic inheritance are commonly conceptualized as transmission of information from one generation to the next [67–71]. Here we show that, from the perspective of a developmental switch, this use of information language can be justified because natural selection on both forms of inheritance, and responses to the non-inherited environment, tends to maximize mutual information between phenotype and selective environment (S1 File). There is no difference between genetic and non-genetic inputs in terms of their effects on development *per se*, but only in the process by which different mechanisms come to carry informational content [35, 36]. Many researchers understandably and rightly focus on the different mechanisms of inheritance (e.g. genetic versus non-genetic inheritance). This focus can obscure the commonality in the way the mechanisms have an effect on development: they all act so as to bias development in one way or another, and the value of doing so depends on the information they carry. This point is very straightforward, almost trivial, when one takes an evolutionary and informational perspective on the problem (i.e., the problem of having a developmental system that unfolds appropriately in different circumstances). It is by no means obvious when one takes a mechanistic approach, which is also important and valuable in itself. This difference in perspective may partly explain why non-genetic inheritance has proved controversial. Our approach helps to make explicit how different mechanisms can have adaptive value.

Our first example concerns the evolution of maternal effects on germination strategies in spatially heterogeneous environments. While our model is parameterized to a spatially varying environment, the conclusions also apply to a situation of temporally varying environmental change with stochastic switching [46]. In support of verbal arguments (e.g. [52]), the autocorrelation between maternal and offspring environment is a key requirement for maternal effects to evolve. Autocorrelation is likely to be quite high in many sedentary organisms like plants and is also an intrinsic feature of many seasonal environments. However, the relationship between maternal environment or phenotype and offspring environment is rarely quantified in studies of maternal effects, and low realised correlation may be one reason for the limited empirical evidence for adaptive transmission of information between generations [72, 73]. Given that the autocorrelation of maternal and offspring environments is perhaps the single most important factor for the evolution of anticipatory parental effects, systems that can be robustly identified to differ in this respect provide outstanding opportunities for testing the theory. This includes species that live in environments with different degrees of seasonality or where the timing of breeding is more or less coupled to reliable environmental cues, such as photoperiod. An additional important aspect in this regard is the distribution of environments since adaptive, non-genetic responses to rare environments are less likely to evolve [74]. This is evident in our model where maternal effects do not evolve, and hence morph type is determined by a genetic locus, under modest seed dispersal when one environment is encountered by only 10% of individuals per generation. This frequency of environments appears to be realistic for many organisms, and we suggest that future empirical work needs to pay more attention to the rate at which different environments are encountered when making predictions about the extent of maternal determination of offspring phenotype (the strength of selection in each environment and environment-specific dispersal rates are also important factors to consider [73]).

Genetic divergence between patches in meta-populations is often limited because of pollen or sperm dispersal. As expected, this also prevents strong genetic effects on the timing of seed germination in our plant example. Pollen dispersal also prevents the evolution of paternal effects because it reduces the autocorrelation between the paternal and offspring environment. It is important to note that with the life history modelled here, the paternal phenotype only carries information about the offspring environment when seed dispersal also is low. This reduces the overall importance of information transmitted through the male germ line, although the added information value may be sufficiently high to select for paternal effects under some circumstances (e.g., when the additive effect of both sources of input is sufficiently high or when fathers, but not mothers, show environment-specific phenotypes, which was not modelled here). There are empirical examples of putative adaptive paternal effects [75, 76], and this model framework would allow assessment of whether or not the life history and environmental context in these cases are consistent with such predictions.

Non-genetic transmission of information between generations also readily evolves in a temporally variable environment. The same general criteria, for example, the extent to which parental phenotypes correlate with the selective agent in the following generation, apply here. Transmission of epigenetic variants down generations has received much attention and we show that increased stability is favoured when environmental change is infrequent relative to generation time and the information content of the parental phenotype is low (Fig. 5). Other candidate systems that may exhibit semi-stable non-genetic inheritance are those where the environment remains stable for tens of generations and where the parental phenotype at the time of reproduction is similar across environments (thereby providing limited information), which may apply to organisms in patchy or seasonal environments (the frog meta-population described in [77] could be one example). Although direct genetic input is not considered in this model, it is unlikely it would have a major effect as we do not consider periods of environmental stability long enough to favour the build up of genetic information over parental effects or offspring plasticity.

In the locust example, we also consider an alternative mechanism of non-genetic inheritance, that of phenotype-dependent transmission stability of epigenetic states. Research on DNA methylation and other epigenetic mechanisms suggests that transmission stability could depend on the phenotype of the parents. For example, in mice, the extent to which offspring inherit methylation status of the agouti locus (with consequences for coat colour, obesity and diabetes) depends on the phenotype of their mother [78]. In the locust example, this mechanism appears sufficient to generate differences in the transgenerational rate of transition between phases when the reliability of cues or strength of selection varies between environments. However, the parameter space that resulted in such hysteresis effects was quite restricted and other mechanisms, such as within-generation behavioural responses to population density [59, 60], could produce a better fit to the data.

Although we demonstrate that non-genetic inheritance can be adaptive across a range of conditions, most of these rely on limited availability of direct environmental cues (or inability, or cost associated with responding to these cues). Thus, when both parental and environmental cues are available, the contribution of non-genetic inheritance to morph determination is likely to be quite minor. The most likely candidates for parental determination of offspring phenotype are therefore those where direct cues are unavailable or costly to assess, environments at the time of phenotype determination are not a good predictor of the environment of selection, when phenotypes are expressed at a stage in development where the necessary sensory systems for processing environmental information are lacking, or when a substantial time is required from assessing the cue to develop the phenotype [13]. Offspring diapause is one example that is likely to fit these criteria well and comparative studies of species with primarily maternal versus environmental determination of diapause [79, 80] would be good systems to test these predictions.

Both examples in this paper are relatively simple and there are a number of interesting additional details that apply to many biological systems. For example, the maternal phenotype does not only affect offspring development directly by providing resources or information, but may also contribute indirectly by modifying offspring environment [5]. Such effects can easily be included in this model framework (see Fig. 1). It is also possible to allow earlier generations to contribute to development, for example by cumulative modification of the external environment [5]. Another important aspect that we excluded from our examples is the evolution of the parental phenotype itself. There are cases where selection can modify the expression of parental phenotype to transmit more information to offspring [17, 27, 36]. It is also possible that discordant selection across generations will reduce information transfer [81, 82]. Such scenarios could be implemented with this model approach.

In summary, we have expanded on a previous model of within-generation plasticity [35] to generate a common framework for addressing the adaptive significance of different forms of non-genetic inheritance. We show how both genetic and non-genetic inheritance can be expressed in terms of information and how adaptive evolution of phenotype determination in this framework can be captured in informational terms. We put this framework to use to derive predictions for when non-genetic inheritance of polymorphisms are advantageous over genetic and environmental determination. Parental morph determination evolves under both conditions of temporal and spatial environmental change, and the model identifies key variables that should covary with the mechanism of morph determination, which provides opportunities for experimental and comparative tests.

## Supporting Information

S1 File Adaptive evolution of developmental switches in terms of mutual information. Simplified analytical version of the framework presented in the paper which predicts when genetic, parental and offspring inputs to development will evolve depending on how mutual information is maximized.
(DOCX)

S2 File Full simulation results from plant and locust models. Complete details on the parameters used in the simulation models, including description and figures of the results for all parameter combinations tested and example trajectories indicating stability of evolved locus values.
(DOCX)
