# Supplementary material for: The Information Value of Non-Genetic Inheritance in Plants and Animals
Source: PLoS One. 2015 Jan 20;10(1):e0116996. doi: 10.1371/journal.pone.0116996 (PMC4300080; doi:10.1371/journal.pone.0116996)
Supplement: S1 File — Simplified analytical version of the framework presented in the paper which predicts when genetic, parental and offspring inputs to development will evolve depending on how mutual information is maximized. (DOCX) [file pone.0116996.s001.docx]

**File S1: Adaptive evolution of developmental switches in terms of mutual information**

**Supplementary information for:**

**The information value of non-genetic inheritance in plants and animals**

Sinead English^1^, Ido Pen^3^, Nicholas Shea^2^ & Tobias Uller^1,4,5^

1. Edward Grey Institute, Department of Zoology, University of Oxford, OX1 3PS, Oxford, UK. Tel: +441865281194, email: sineadenglish@cantab.net

2. Theoretical Biology Group, Centre for Ecological and Evolutionary Studies, University of Groningen, PO Box 11103, 9700CC Groningen, the Netherlands. Tel: +31503638083, email: i.r.pen@rug.nl

3. Department of Philosophy, King's College London Strand, London WC2R 2LS , UK. Tel: +442078482893, email: nicholas.shea@kcl.ac.uk

4. Department of Biology, University of Lund, Sölvegatan 37, SE 223 62 Lund, Sweden

5. Corresponding author: tobias.uller@zoo.ox.ac.uk

**Background**

Heredity and evolution are often considered in informational terms [1,2]. On the one hand, natural selection on heritable phenotypic variation is an ‘information-generating’ process, by which differential survival and reproductive success of phenotypes transfers information from the environment into the hereditary particles, i.e., the genes [3,4]. It has recently been shown formally that the information accumulation of replicating entities under natural selection maximizes Fisher information about the environment [4–6]. An alternative view, largely motivated by research in behavioural and evolutionary ecology, is to focus on how individual organisms maximize the use of information in their environment [7,8], including information transmitted from parents [9–12].

Leimar and colleagues [13,14] showed that both inherited genes and the external environment can be seen as sources of information that can be capitalized upon by developmental mechanisms. Shea et al. [15] extended this to also consider non-genetic inheritance (see also [16]). In the model presented in the main paper, we follow these authors by considering the evolution of responsiveness to different sources of inherited and non-inherited inputs from the perspective of a developmental switch. In this supplement, we show that this approach also has an attractive link to information theory because adaptive evolution of the developmental switch maximizes mutual information between phenotype and selective context.

**Model properties**

We use the model presented in Shea et al. [15] (Figure A), which is an extension of a model originally presented by Leimar et al. [13] and a simple and idealized version of the general framework presented in the main paper (Figure 1). The rationale of the approach is that all three sources of input – genetic, environmental, and parental – that contribute to the liability of the switch can correlate with the selective agent following morph determination (as explained in the main text and Figure 1).

Consider a population consisting of two equally large subpopulations in environments E_1_ and E_2_, connected by a low rate of migration *d* (the probability an individual permanently moves between the two subpopulations before reproducing). The life history follows a simple structure with non-overlapping generations: Reproduction → Development → Migration → Selection → Reproduction. Thus, given a low migration rate *d*, the environment of development is positively correlated with the environment of selection. Organisms are haploids and can develop two phenotypes, P_1_ and P_2_. In E_1_, the optimal offspring phenotype is P_1_, in E_2_ it is P_2_. Non-matching phenotypes have relative fitness 1-*s*. Migration is independent of phenotype (Figure A).


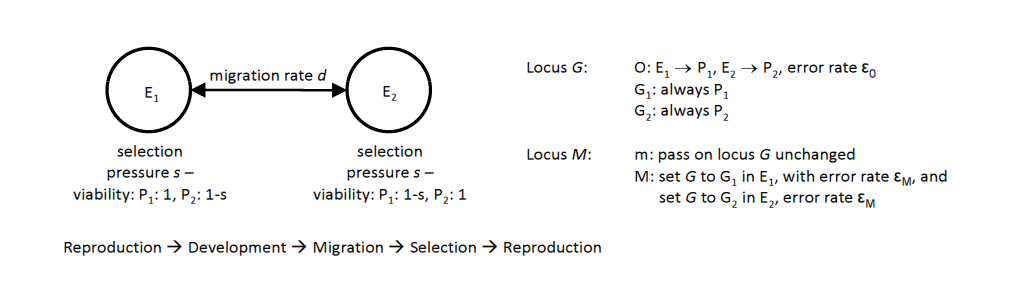


**Figure A. Summary of the model.** A population consisting of individuals of two different phenotypes, P_1_ and P_2_, live in an environment that can occur in two different forms, E_1_ and E_2_. Migration between patches occurs at rate *d* and selection against non-matching phenotypes is s.

Organisms have a genetic locus A, with three different possible states (alleles). When in possession of allele O offspring produce an environment-specific phenotype by detecting their natal environment with error rate *ε*_O_ (the probability of identifying the natal environment as E_1_ when it is in fact E_2_, and vice versa) and develop the matching phenotype accordingly. When in possession of allele G*_i_* ($i\in\{1,2\}$) offspring always develop phenotype P*_i_*, regardless of their natal environment. There is a second locus, B, the *maternal effect locus* with alleles m and M. Allele m has no effect, whereas M results in an adult marking its offspring’s locus A with G*_i_* when the adult detects itself to be in E*_i_* (with error probability $\varepsilon_{\text{M}}$).

Thus, there are three potential sources of information for offspring when dispersal rates are low. First, if an offspring organism carries O, it can extract information about the future selective regime from its environment of development. Second, in the absence of maternal effects, selection can build up differences in allele frequencies in the two environments because G_1_ is favored in E_1_ and G_2_ is favored in E_2_. Hence G_1_ and G_2_ carry information as a result of past selection; if an offspring receives G_1_ from its mother it is more likely to be developing in E_1_, and then to be subject to selection in E_1_, than if it receives G_2_ and vice versa. Third, in the presence of maternal effects (mothers carrying the M allele) the G*_i_* alleles also provide information about an offspring’s maternal environment.

**Results and Connection to Mutual Information**

We first briefly summarize the results of Shea et al. [15]. Suppose the population is fixed for m and O. Then after migration, the frequency of matching phenotypes (p_O_) is

(1)

If the population is fixed for m and there are no O alleles, then G_1_ and G_2_ reach equilibrium frequencies such that the frequency of matching phenotypes (p_G_) equals

(2)

Shea et al. [15] showed that there is no equilibrium where O-alleles and G-alleles stably co-exist, and that O-alleles will go to fixation if

(3)

Conversely, if , then O-alleles will disappear from the population, i.e., relying on a genetic cue leads to a higher equilibrium number of well-adapted individuals.

Similarly, in a population fixed for the M allele, the frequency of matching phenotypes (p_M_) equals

(4)

It turns out that M goes to fixation whenever

. (5)

In other words, the source of information that offspring ultimately rely on is that source which yields the highest frequency of matching phenotypes.

Here we show that the same result can also be expressed in terms of the concept of mutual information. Mutual information *I* describes the extent to which an observation of one random variable reduces uncertainty about another random variable in terms of entropy [17]. In general, for discrete random variables *X* and *Y*, *I* is defined as

(6)

where *p_xy_* , *p_x_* and *p_y_* are the joint and marginal distributions of realizations of *X* and *Y*. For our purpose we write *I_G_* for the mutual information of the genetic locus *G* relative to *E*, and *I_O_* for the mutual information of relative to *E*, where is the environment observed by an offspring. The joint and marginal distributions of *G* and *E* in the G-equilibrium are given by Table A.

Table A.

|  | E_1_ | E_2_ | *p_G_* |
| --- | --- | --- | --- |
| G_1_ |  |  | ½ |
| G_2_ |  |  | ½ |
| *p_E_* | ½ | ½ |  |

Therefore

(7)

Likewise, in the O-equilibrium we have the distributions in Table B.

Table B.

|  | E_1_ | E_2_ | $p_{\tilde{E}}$ |
| --- | --- | --- | --- |
|  |  |  | ½ |
|  |  |  | ½ |
| *p_E_* | ½ | ½ |  |

and the mutual information is now given by

(8)

Since the function $f(x)=1+x\log x+(1-x)\log(1-x)$ is strictly increasing on the interval, it follows that

(9)

Similarly, it can be shown that selection favors maternal modification of G-alleles if and only if this increases the associated mutual information.

The result above demonstrates a connection between mutual information and fitness in this model, but the connection is local rather than general. A developmental switch that minimized fitness, leading the organism to develop precisely the wrong phenotype in each environment, would also maximize mutual information between phenotype and selective regime. So while natural selection arrives at a local maximum of mutual information, it will not generally be true that mutual information only is maximized in the region of phenotype space favored by selection.

**One final point**

In the philosophical literature the idea that genes transmit information down the generations in any substantive sense remains controversial [18–20]. It is much less controversial that when an organism is designed to make use of a signal or a cue it is making use of special subclass of correlational information, which we might call functional information. Shea et al. [15] showed that genetic and non-genetic inheritance and environmental cues are relied on interchangeably. The demonstration that all these sources of information are being relied upon to maximize the mutual information between phenotypes and the environment for adaptive purposes underlines the point that the correlational information generated by natural selection, which inheritance systems are designed to transmit, can be seen as a special case of semantic, functional information. Evolution of inheritance therefore resembles evolution of signaling systems [21,22].

**References**

1. Adami C (2012) The use of information theory in evolutionary biology. Ann N Y Acad Sci 1256: 49–65. doi:10.1111/j.1749-6632.2011.06422.x.

2. Maynard-Smith J (2000) The Concept of Information in Biology. Philos Sci 67: 177–194.

3. Kimura M (1961) Natural selection as the process of accumulating genetic information in adaptive evolution. Genet Res 2: 127–140.

4. Frank SA (2009) Natural selection maximizes Fisher information. J Evol Biol 22: 231–244. doi:10.1111/j.1420-9101.2008.01647.x.

5. Harper M (2009) Information Geometry and Evolutionary Game Theory. arXiv:09111383v1.

6. Harper M (2010) The Replicator Equation as an Inference Dynamic. arXiv:09111763v3.

7. Dall SRX, Giraldeau L-A, Olsson O, McNamara JM, Stephens DW (2005) Information and its use by animals in evolutionary ecology. Trends Ecol Evol 20: 187–193. doi:10.1016/j.tree.2005.01.010.

8. Schmidt KA, Dall SRX, van Gils JA (2010) The ecology of information: an overview on the ecological significance of making informed decisions. Oikos 119: 304–316. doi:10.1111/j.1600-0706.2009.17573.x.

9. Jablonka E (2002) Information: Its Interpretation, Its Inheritance, and Its Sharing. Philos Sci 69: 578–605.

10. Jablonka E, Lamb MJ (2014) Evolution in Four Dimensions: Genetic, Epigenetic, Behavioral, and Symbolic Variation in the History of Life. 2nd ed. Cambridge MA: MIT Press.

11. Danchin É, Charmantier A, Champagne FA, Mesoudi A, Pujol B, et al. (2011) Beyond DNA: integrating inclusive inheritance into an extended theory of evolution. Nat Rev Genet 12: 475–486. doi:10.1038/nrg3028.

12. Danchin E (2013) Avatars of information: towards an inclusive evolutionary synthesis. Trends Ecol Evol 28: 351–358. doi:10.1016/j.tree.2013.02.010.

13. Leimar O, Hammerstein P, Van Dooren TJM (2006) A new perspective on developmental plasticity and the principles of adaptive morph determination. Am Nat 167: 367–376. doi:10.1086/499566.

14. Leimar O (2009) Environmental and genetic cues in the evolution of phenotypic polymorphism. Evol Ecol 23: 125–135. doi:10.1007/s10682-007-9194-4.

15. Shea N, Pen I, Uller T (2011) Three epigenetic information channels and their different roles in evolution. J Evol Biol 24: 1178–1187. doi:10.1111/j.1420-9101.2011.02235.x.

16. Bergstrom CT, Rosvall M (2011) The transmission sense of information. Biol Philos 26: 159–176. doi:10.1007/s10539-009-9180-z.

17. Shannon CE (1949) The mathematical theory of communication. In: Shannon CE, Weaver W, editors. The Mathematical Theory of Communication. Urbana: University of Illinois Press.

18. Godfrey-Smith P (2007) Innateness and genetic information. In: Carruthers P, Laurence S, Stich S, editors. The Innate Mind: Foundations and the Future. Oxford/New York: Oxford University Press. pp. 55–68.

19. Griffiths PE (2001) Genetic Information: A Metaphor in Search of a Theory. Philos Sci 68: 394–412.

20. Oyama S (1985) The Ontogeny of Information: Developmental Systems and Evolution. Cambridge: Cambridge University Press.

21. Skyrms B (2010) Signals. Evolution, learning and information. New York: Oxford University Press.

22. Shea N (in press) Two Modes of Transgenerational Information Transmission. In: Calcott B, Joyce R, Sterelny K, editors. Signaling, Commitment and Emotion. Cambridge MA: MIT Press.
