# Supplementary material for: The Information Value of Non-Genetic Inheritance in Plants and Animals
Source: PLoS One. 2015 Jan 20;10(1):e0116996. doi: 10.1371/journal.pone.0116996 (PMC4300080; doi:10.1371/journal.pone.0116996)
Supplement: S2 File — Complete details on the parameters used in the simulation models, including description and figures of the results for all parameter combinations tested and example trajectories indicating stability of evolved locus values. (DOCX) [file pone.0116996.s002.docx]

**File S2: Full simulation results from plant and locust models**

**Supplementary information for:**

**The information value of non-genetic inheritance in plants and animals**

Sinead English^1^, Ido Pen^3^, Nicholas Shea^2^ & Tobias Uller^1,4,5^

1. Edward Grey Institute, Department of Zoology, University of Oxford, OX1 3PS, Oxford, UK. Tel: +441865281194, email: sineadenglish@cantab.net

2. Theoretical Biology Group, Centre for Ecological and Evolutionary Studies, University of Groningen, PO Box 11103, 9700CC Groningen, the Netherlands. Tel: +31503638083, email: i.r.pen@rug.nl

3. Department of Philosophy, King's College London Strand, London WC2R 2LS , UK. Tel: +442078482893, email: nicholas.shea@kcl.ac.uk

4. Department of Biology, University of Lund, Sölvegatan 37, SE 223 62 Lund, Sweden

5. Corresponding author: tobias.uller@zoo.ox.ac.uk

**Model 1: Maternal effects on seed germination**

In our first model, we investigate the conditions favouring maternal effects, compared to genetic, paternal and direct offspring effects, on timing of germination in a plant system. Details on the life history of the system are provided in the main text. Table C describes all the parameters used in the model and their values when fixed or varied depending on the specific question addressed. We assessed that the loci had reached stable values through visual assessment of the trajectories for average loci values across generations (see example graphs in Figure N).

**Table C. Plant model overview.** Description of all loci and parameters varied in the simulations for Model 1. Columns 1.1 ­– 1.4 refer to the relevant sub-models (as described in sections with the same heading) and X denotes whether a locus evolved or a parameter was varied for that particular model.

| **Description** | **Name** | **1.1** | **1.2** | **1.3** | **1.4** | **Fixed** | **Varied** |
| --- | --- | --- | --- | --- | --- | --- | --- |
| *Loci* |  |  |  |  |  |  |  |
| Genetic input | *y_G_* |  |  | X | X |  |  |
| Maternal weighing | *b_M_* | X | X | X | X |  |  |
| Paternal weighing | *b_F_* | X |  |  |  |  |  |
| Offspring weighing | *b_O_* |  | X |  | X |  |  |
| *Parameters* |  |  |  |  |  |  |  |
| Seed dispersal | *d* | X |  | X | X | 0.1 | 0, 0.1, 0.5, 0.9, 1 |
| Pollen dispersal | *q* | X |  |  |  | 0.9 | 0, 0.1, 0.5, 0.9, 1 |
| Patch frequency | *f* | X |  | X | X | 0.5 | 0.1, 0.5 |
| Parent phenotype | *σ_P_* |  | X | X | X | 0.1 | 0.01, 0.1, 0.5, 1, 5 |
| Offspring cue | *σ_O_* |  | X |  | X | 0.1 | 0.01, 0.1, 0.5, 1, 5 |
| *Constants (all models)* |  |  |  |  |  |  |  |
| Selection | *s* |  |  |  |  | 0.1 |  |
| No. patches | *Npatch* |  |  |  |  | 200 |  |
| No. adults per patch | *Nadult* |  |  |  |  | 100 |  |
| No. generations | *Ngen* |  |  |  |  | 200000 |  |
| Mutation step | *mu* |  |  |  |  | 0.001 |  |
| SD mutation step | *sdmu* |  |  |  |  | 0.005 |  |
| No. simulation runs | *Nsim* |  |  |  |  | 10 |  |

**1.1 Under what conditions of seed and pollen dispersal do maternal versus paternal effects evolve, and how does this depend on the frequency of patches?**

In this model, the loci *y_G_* and *b_O_* are fixed at zero and offspring only weigh environment-specific parental phenotype (i.e. *b_M_* and *b_F_* evolve). We fix parental environment-trait distribution and offspring error to be relatively reliable (*σ_P_* = *σ_O_* = 0.1), and consider variation in seed and pollen dispersal (*d* and *q* vary between 0 and 1) and whether patches are equally common or if one patch is rare (proportion 0.1 of patches). We predict that maternal effects will evolve under most circumstances, as the only source of information is maternal or paternal effects.

We find, as expected, that maternal effects evolve under most circumstances, apart from when seed dispersal is 1 and patches are equally distributed. Paternal effects are generally weak or not under selection at all, with the exception when there is zero pollen dispersal (Figure B). When one patch is rare, paternal effects evolve to slightly higher values when seeds disperse with a probability 1 (Figure B).


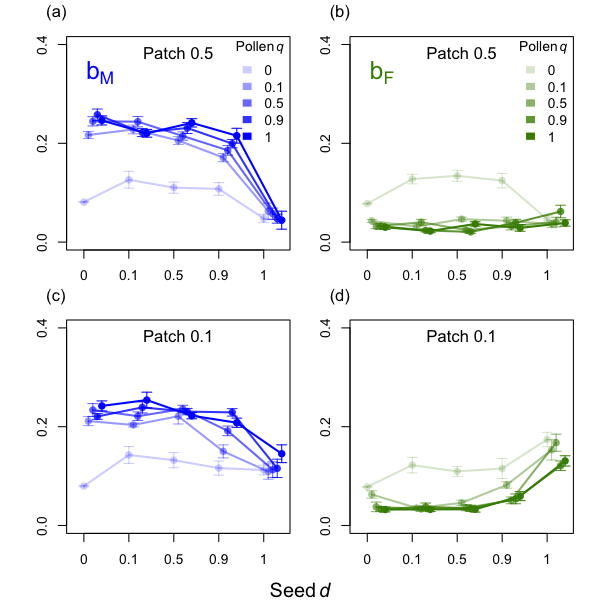


**Figure B. Maternal versus paternal effects.** Effect of pollen (different lines) and seed dispersal (x-axis) on weighting of maternal (b_M,_ blue) or paternal (b_F_, green) phenotype depending on patch frequency (upper versus lower plots). Shown are mean ± SE evolved values for last 20,000 generations across 6 runs.

**1.2 How does variation in parent phenotype distribution and offspring error affect whether maternal versus direct offspring assessment evolve?**

Here, only maternal and offspring weighting evolve (*b_M_* and *b_O_),* while *y_G_* and *b_F_* are fixed. Based on results above and biological intuition of the system, we fix seed dispersal at 0.1, pollen dispersal at 0.9 and patch frequency at 0.5. We vary parental environment-trait distribution, *σ_P_* and offspring environment-cue distribution, *σ_O_* (five levels between 0.01 and 5) to examine how maternal effects evolve in response to the information value of maternal and offspring environmental inputs.

We find that offspring are selected to weigh their own estimated environment rather than their mother's phenotype under most conditions (Figure C). This is because even with a small amount of dispersal (0.1), all else being equal, offspring gain more information through direct environmental effects rather than those from the previous generation. Thus, it is only when direct environmental cues are relatively uninformative that maternal effects evolve (Figure C), i.e. when the offspring environment cue is unspecific (high *σ_O_*) and, under these circumstances, when maternal phenotypes are environment-specific (low *σ_P_*; Figure C)..

For each parameter combination, we also calculated the proportion of total variance explained by offspring and maternal environment (termed R*^2^*_O_ or R*^2^*_M_ respectively), to reflect empirical approaches to measuring parental versus offspring effects. These were calculated using type III sums of squares produced by ANOVA, values provided by the drop1 function applied to a lm model in the 'stats' package. Maternal environment explains a greater proportion of variance when offspring error is high and there is little overlap in parental phenotype between environments; and offspring phenotype explains nearly all of the variance when offspring error is low, regardless of how well the parental phenotype reflects the environment (Figure C).


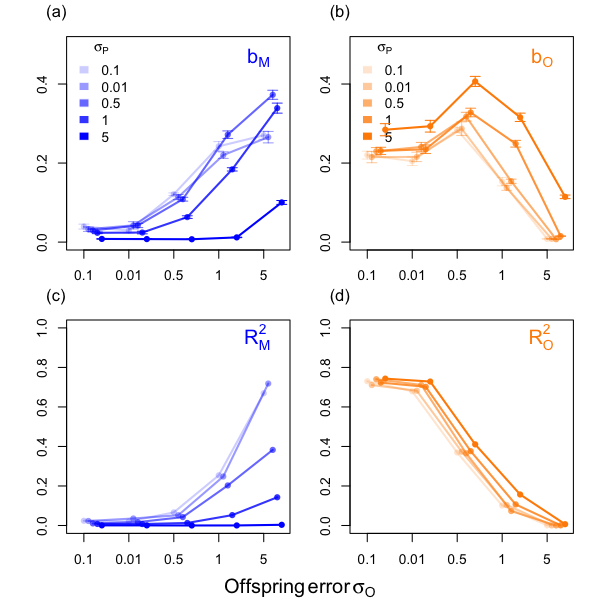


**Figure C. Maternal versus direct environment effects.** Effect of parental trait-environment distribution (*σ_P_*, different lines) and offspring cue-environment distribution (*σ_O_*, x-axis) on weighing of maternal (blue) or offspring (orange) input to phenotype. Shown are (a-b) evolved values of weighting loci, b_M_ and b_O_ (mean±SE of values for last 20,000 generations across 6 runs); and (c-d) proportion of variance in trait liability *y* explained by maternal or offspring environment (i.e., R*^2^*_M_ or R*^2^*_O_ respectively).

**1.3 How do seed dispersal and patch frequency determine whether maternal effects or genetic inheritance evolve?**

In these simulations, we allow *y_G_* and *b_M_* to evolve, fixing *b_F_* and *b_O_* at zero. We also fix values of pollen dispersal at 0.9 and consider how variation in seed dispersal (between 0 and 1) and environment-specific parent-trait distribution, *σ_P_* (between 0.01 and 5) affect the evolution of genetic versus maternal input to morph determination, depending on whether patches are equal or one is rare. We expect genes to be informative when the maternal phenotype is very uninformative and when seed dispersal is very low (if selection builds up differences in allele frequencies between patches, although selection is weak in these models). If one patch is rare, and seed dispersal is high, we expect genetic effects to evolve as producing a single phenotype should produce a higher overall fit than responding to maternal environmental effects.

We find that, when patches are of equal frequency, weighing of maternal input always evolves unless maternal distribution is highly overlapping (Figure D). When one patch is rare, genetic input evolves in the opposite direction to weighing of the maternal effect (Figure D) and is strongest ­– and there is no maternal effect – when maternal phenotype is not very environment-specific (high *σ_P_*). *y_G_* evolves to negative values under these settings because the most common environment in our model formulation is type 0 (shade), i.e. favouring a negative liability. When patches are equal, the proportion of matching phenotypes produced under high seed dispersal and/or high maternal-environment distribution is 0.5, consistent with random morph determination; whereas if one environment is rare, then the proportion of matching phenotypes produced under these conditions (i.e. when morph is predominately determined by genetic input) is approximately 0.9, as the phenotype matching the most common environment is produced nearly all of the time.


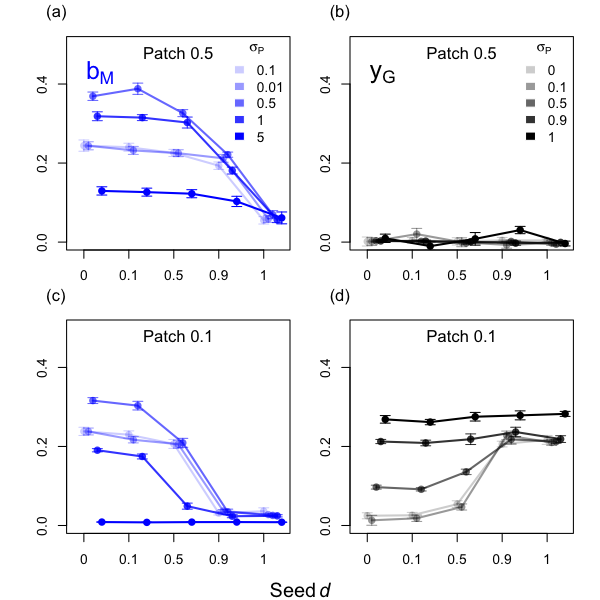


**Figure D. Maternal versus genetic effects.** Effect of parent environment-phenotype trait distribution (*σ_P_*) on weighting of maternal (*b_M_*) and genetic input (*y_G_*) depending on patch frequency (upper versus lower plots) and for different values of seed dispersal (x-axis). Levels of *y_G_* are inverted so they are on a comparable scale to *b_M_* (values on the original scale are negative as the most common patch is of the shaded type, for which *y* evolves to negative values).

**1.4 Under what general conditions do maternal, genetic or direct offspring effects evolve?**

In this simulation, we consider the broad conditions under which *y_G_, b_M,_* and *b_O_* evolve while setting *b_F_* to zero (consistent with the rationale for paternal effects being limited in this system described in section 1.1), varying seed dispersal, patch frequency, maternal trait distribution, and offspring error. Pollen dispersal *q* is fixed at 0.9, and we vary all other parameters considered (*d*, *f, σ_P_* and *σ_O_*; see Table A for values). We predict offspring assessment of their own environment (*b_O_*) to be under strongest selection as it is the most reliable input to morph determination under most parameters, apart from when *σ_O_* is high. We expect maternal effects only when offspring cues are uninformative; and given the dispersal rates and weak selection considered, we do not expect alleles to provide much information.

We find that genetic cues only evolve when one patch is rare, when offspring environment-cue overlap *σ_O_* is high and (if seed dispersal *d* is limited) when parent cue specificity is low (high *σ_P_*) (Figure E). Maternal weighting evolves when *σ_O_* is high, and *d* is low (Figure F). Response to environmental cues evolves under most scenarios, apart from when *σ_O_* is high. (Figure G), but is under weaker selection when seed dispersal is low and parental specificity is higher (low *σ_P_*, Figure G).

Figure E


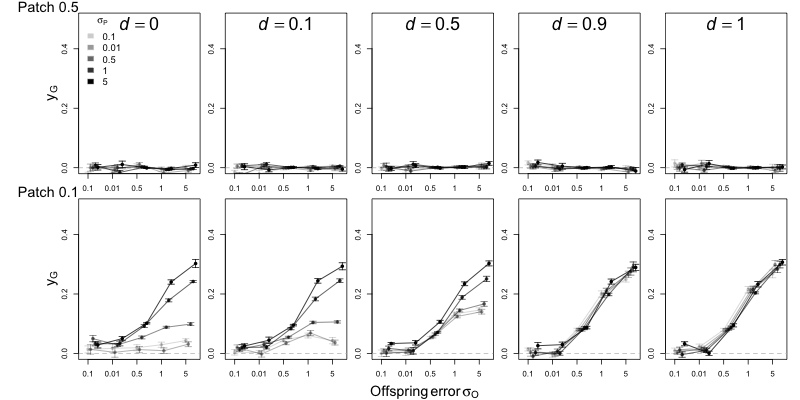


Figure F
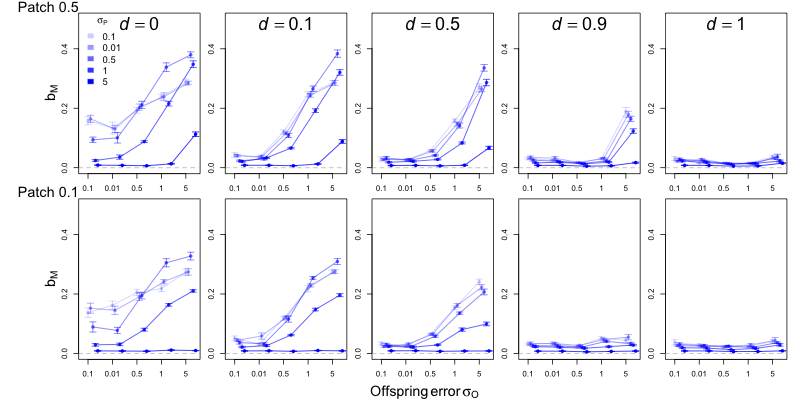


Figure G


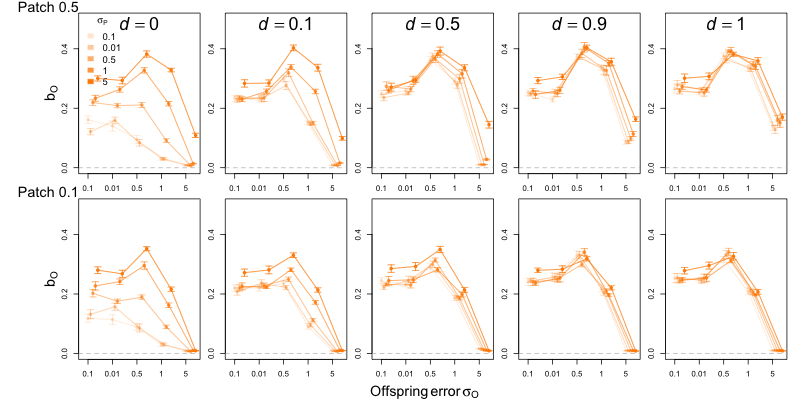


**Figures E-G. General conditions under which genetic, maternal and direct offspring input evolves.** Evolved values of *y_G_* (black), *b_M_* (blue) and *b_O_* (orange) depending on patch frequency (upper and lower panels), seed dispersal (panels from left to right), offspring error (values on x-axis) and parent specificity (different lines). Levels of *y_G_* are inverted so they are on a comparable scale to *b_M_* (as explained above).

**Model 2: Transgenerational phase shift in locusts**

In our second model, we investigate the evolution of different forms of non-genetic inheritance ­– direct maternal environment effects versus (partial) inheritance of the previous generation's maternal effect – on phase state in desert locusts, and compare these inputs to offspring assessment of the local environment. Description and details of the system's life history are provided in the main text. Table D describes the parameters investigated and their values when fixed or varied depending on the specific question addressed. We adjusted the mutation rate to be higher than in the Plant model example such that trait values evolved to stable values (see example in Figure O).

**2.1 What is the effect of period of environmental stability and maternal error on the evolution of different forms of non-genetic inheritance?**

In this simulation, we only consider the evolution of non-genetic inheritance (both the direct maternal environment effect *m*, and transmission stability *h*) and set offspring weighing of their own environment (*b_O_*) to zero. Selection against mismatched phenotypes is the same in both environments (*s* = 0.1) and we consider variation in the period of environmental stability *T_U_* = *T_C_* = {5, 10, 15} and in maternal error *em_U_* = *em_C_* = {0.1, 0.2, 0.4}. Based on results of a previous model (Uller & Pen in review), we expect a maternal environment effect to evolve when maternal error is low and the environments are relatively stable across generations; and transmission stability to evolve when maternal error is high and the environments are relatively stable across generations.

**Table D. Locust model overview.** Description of all loci and parameters varied in the simulations for Model 2. Columns 2.1–2.3 refer to the relevant sub-models (as described in sections with the same heading) and X denotes whether a locus evolved or a parameter was varied for that particular model.

| **Description** | **Variable** | **2.1** | **2.2** | **2.3** | **Fixed** | **Varied** |
| --- | --- | --- | --- | --- | --- | --- |
| *Loci* |  |  |  |  |  |  |
| Maternal environment (uncrowded) | *m*_U_ | X | X | X |  |  |
| Maternal environment (crowded) | *m*_C_ | X | X | X |  |  |
| Transmission stability (solitary) | *h*_S_ | X | X | X |  |  |
| Transmission stability (gregarious) | *h*_G_ | X | X | X |  |  |
| Offspring weighting | *be* |  |  | X |  |  |
| *Parameters* |  |  |  |  |  |  |
| Period environment is stable (uncrowded) | *T*_U_ | X | X |  | 15 | {5, 10, 15} |
| Period environment is stable (crowded) | *T*_C_ | X | X |  | 15 | {5, 10, 15} |
| Maternal error (uncrowded) | *em*_U_ | X | X | X | 0.4 | {0.1, 0.2, 0.4} |
| Maternal error (crowded) | *em*_C_ | X | X | X | 0.4 | {0.1, 0.2, 0.4} |
| Offspring error (uncrowded) | *eo*_U_ |  |  | X | 0.4 | {0.1, 0.2, 0.4} |
| Offspring error (crowded) | *eo*_C_ |  |  | X | 0.4 | {0.1, 0.2, 0.4} |
| Selection (uncrowded) | *s*_U_ |  | X |  | 0.05 | {0.05, 0.2} |
| Selection (crowded) | *s*_C_ |  | X |  | 0.05 | {0.05, 0.2} |
| *Constants* |  |  |  |  |  |  |
| No. patches | *Npatch* |  |  |  | 1 |  |
| No. adults per patch | *Nadult* |  |  |  | 100000 |  |
| No. generations | *Ngen* |  |  |  | 200000 |  |
| Mutation step | *mu* |  |  |  | 0.01 |  |
| SD mutation step | *sdmu* |  |  |  | 0.05 |  |
| No. simulation runs | *Nsim* |  |  |  | 10 |  |
|  |  |  |  |  |  |  |

We find that, under these conditions where the only sources of input to morph determination are through maternal effects, the maternal environment effect evolves under all conditions, regardless of maternal error or environmental stability (Figure Ha). Transmission stability evolves when maternal error is high and environments are stable, as predicted (Figure Hc). We do not find a strong effect of transmission stability on the level of maternal environment effect that evolves (Figure Ha versus Figure Hb).

**
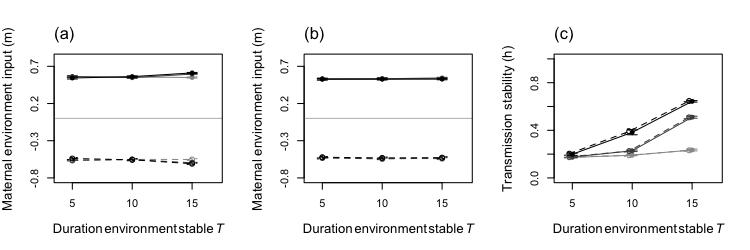
**

**Figure H. Evolution of different modes of non-genetic inheritance.** Effect of environmental stability and maternal error on maternal environment input (a,b) and on transmission stability (c). Values for m are shown for simulations with (a) and without (b) transmission stability. For (a) and (b) solid points and lines denote values for mothers in crowded environments; hollow points and dashed lines denote values for mothers in uncrowded environments; and for transmission stability (c) solid point and lines are for gregarious whereas hollow points and dashed lines are for solitary phenotypes. Shown are evolved values (mean ± SE from last 20,000 generations for 12 simulations)

**2.2 Under what conditions of environment-specific cycle length, maternal error or selection does hysteresis evolve?**

We then extend the simulations above to explore the conditions under which there is a slower transition from a population of predominantly one phenotype to the other phenotype in one direction than in the other (i.e., hysteresis effect, with a more gradual change from gregarious to solitary than from solitary to gregarious populations­, for example) might evolve. In this simulation, *b_O_* does not evolve and we consider three potential asymmetries which might result in this hysteresis: first, we consider asymmetric environmental change, where {*T_U_; T_C_* } = {15, 5} or {5, 15} compared to a baseline of {15, 15}. Second, we consider asymmetries in maternal error in assessing the environment, i.e. {*em_U_; em_C_* } = {0.4, 0.1} or {0.1, 0.4} compared to a baseline of {0.4, 0.4}. Finally, we consider asymmetries in the cost of exhibiting a mismatched phenotype between the environments (it may, for example, be more risky to be a colourful gregarious morph in uncrowded conditions than a cryptic solitary morph in crowded conditions), hence selection varying as {*s_U_; s_C_*} = {0.2, 0.1} or {0.1, 0.2} compared to the {0.1, 0.1} baseline. We predict the environment-specific maternal environment input and phenotype-specific transmission stability to evolve when there are asymmetries in environmental change, maternal error and selection. This could then result in a hysteresis effect although we did not *a priori* predict which of these factors would have the strongest effect on a hysteresis evolving.

We find that with asymmetric environmental stability, environment-specific maternal input evolves to the same value for both environments and at the value of the environment which has longer cycles (Figure Ia). Under these conditions, no transmission stability evolves (Figure Ic) since the environment switches too frequently (Figure Ic). When there is asymmetric maternal error or selection, the maternal environment effect evolves to different absolute values (higher in the environment where error is higher or selection is stronger, respectively, Figure Ia) and transmission stability evolves for both gregarious and solitary phenotypes, but more quickly and to higher levels for the phenotype adapted to the environment in which maternal error is higher or selection is stronger, respectively (Figure Ic). When there is no transmission stability, there is no asymmetry in the level of maternal environment effect (for the simulation where maternal error is asymmetric) and the maternal environment effect evolves to the levels where selection is stronger (for the simulation where selection is asymmetric) (Figure Ib).


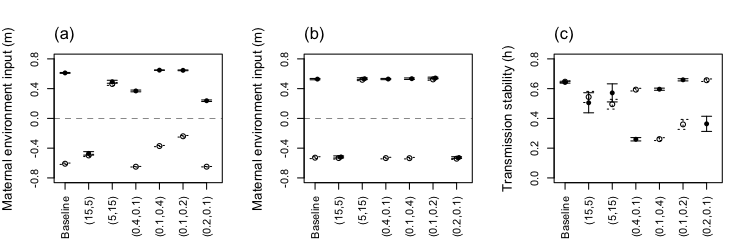


**Figure I. Conditions under which hysteresis effects evolve.** Effect of asymmetric period, maternal error and selection on maternal environment (a-b) and transmission stability (c). Values for m are shown for simulations with (a) and without (b) transmission stability. Shown are evolved values (mean ± SE from last 20,000 generations for 12 simulations) in the uncrowded (hollow circles) and crowded (solid circles) environment.

This effect of asymmetric maternal error on maternal environment effect and transmission stability results in hysteresis. In general, the average number of generations it takes for the proportion of matched phenotypes to stabilise is slightly shorter comparing an environmental switch in one direction to the other (Figure Jb and Jc). The hysteresis effect only evolves when transmission stability is allowed (Figure Jb-c versus Figure Jd-f). Similar results emerge from asymmetric selection, with the change in phenotype following an environmental switch occurring more slowly in one direction than the other (Figure Ab,c). In this case, however, the proportion of matched individuals is much higher in the environment with stronger selection.


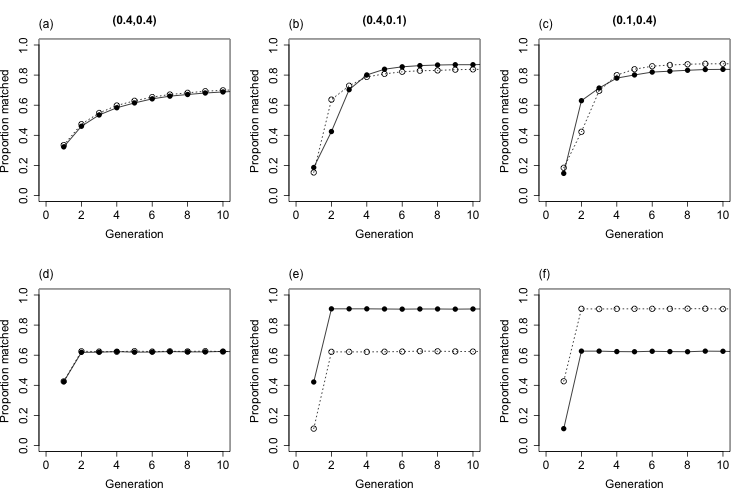


**Figure J. Example simulations to illustrate hysteresis when maternal error is asymmetric.** Proportion of matched individuals following an environmental switch, i.e. from crowded to uncrowded (open circles, dashed lines) or vice versa (solid circles, black lines); depending on asymmetry in maternal error (see graph titles) and whether there is also tranmission stability (a-c) or not (d-f). Shown are example trajectories for one switch incident from one simulation run.


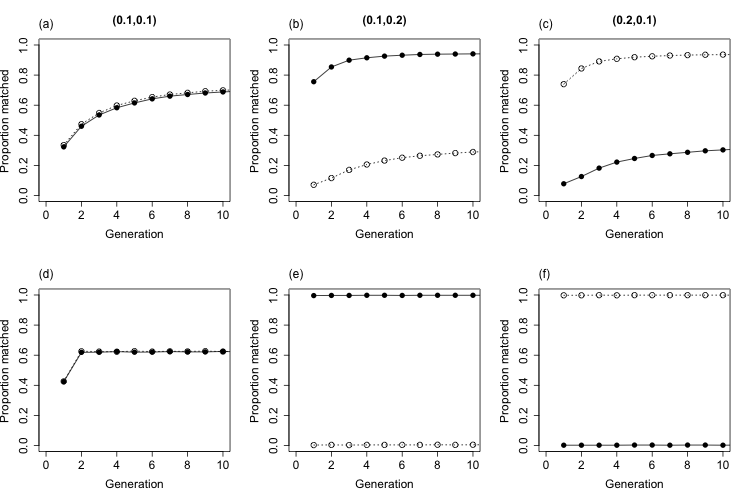


**Figure K. Example simulations to illustrate hysteresis when selection is asymmetric.** Proportion of matched individuals following an environmental switch, i.e. from crowded to uncrowded (open circles, dashed lines) or vice versa (solid circles, black lines); depending on asymmetry in selection (see graph titles) and whether there is also tranmission stability (a-c) or not (d-f). Shown are example trajectories for one switch incident from one simulation run.

**2.3 Does non-genetic inheritance evolve if offspring can assess their own environment?**

We use the simulation model to investigate the conditions under which non-genetic inheritance evolves when within-generation plasticity (which is a feature of the locust system) is allowed. Using results from above, we consider parameter values for when transmission stability evolves (i.e. *s_U_ = s_C_ =* 0.1; *T_U_ = T_C_* = 15 and *em_U_* = *em_C_* = 0.4); and allow offspring assessment of the environment (*b_O_*) to evolve, with varying levels of offspring error considered (*eo_U_* = *eo_C_* = {0.1, 0.2, 0.4}). We expect that offspring will only use maternal cues if offspring error is high. Indeed we find that if offspring assessment is also allowed to evolve, maternal environment effects and transmission stability only evolve when offspring assessment of their own environment is inaccurate (*eo_i_* = 0.4) (Figure L).

We also assess the effect of direct offspring assessment on the hysteresis effect by repeating simulations under conditions of hysteresis (i.e. asymmetric maternal error) but allowing offspring direct assessment with high error (0.4) in both environments. We find that hysteresis still occurs although, with direct offspring assessment, there is no longer a difference in the absolute proportion of individuals who exhibit a matched phenotype in each environment once levels have stabilized (Figure M).


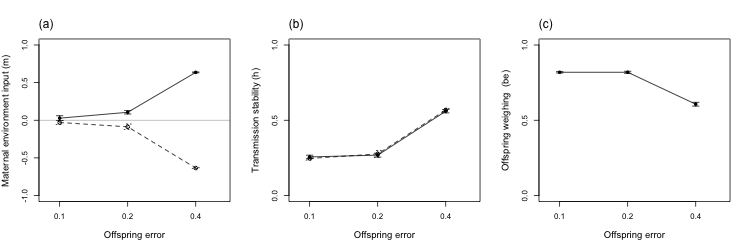


**Figure L. The effect of offspring plasticity on non-genetic inheritance.** Evolution of maternal effects and transmission stability when direct offspring effects also allowed, with open points, dashed lines indicating uncrowded environments (a) and solitary phenotypes (b), whereas solid points and lines indicate crowded environments (a) and gregarious phenotypes (c).


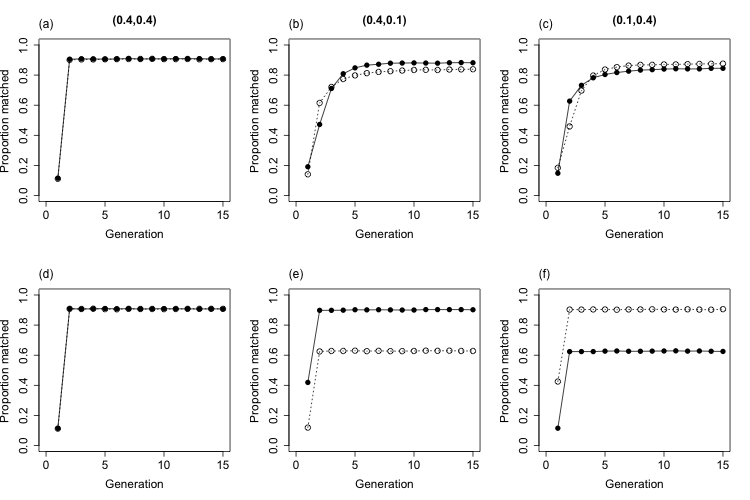


**Figure M. Conditions for hysteresis when offspring are plastic.** Change in the proportion of matching phenotypes after an environmental switch for conditions when offspring assessment is also allowed (headings show maternal error in each environment). Shown are example trajectories for one switch incident from one simulation run.


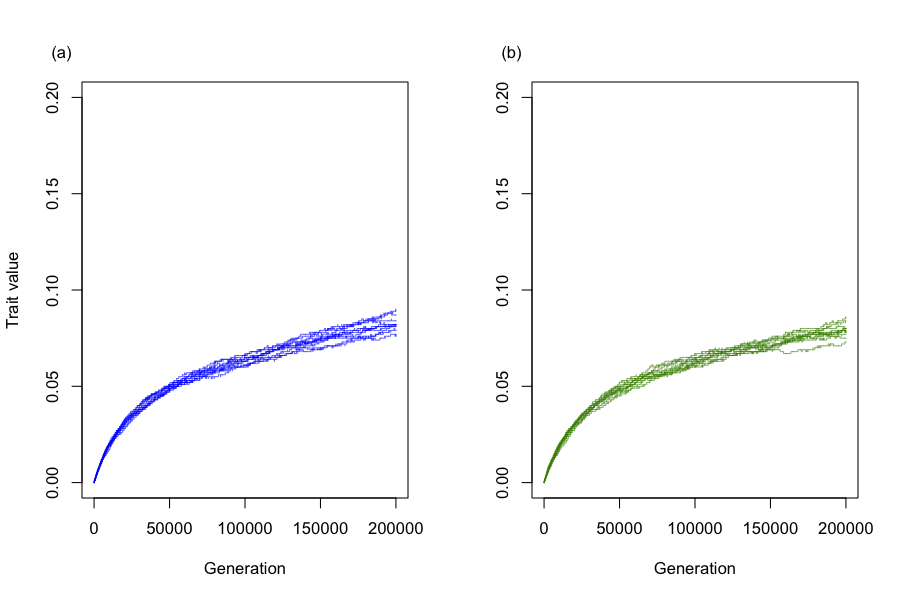


**Figure N.** Example trajectories of simulation runs over time for (a) maternal input, *b_M_* (blue) and (b) paternal input, *b_F_* (green) for the first combination of simulation parameters in the plant model example 1.1, i.e. when seed dispersal is 0, pollen dispersal is 0, parent and offspring error are 0.1 and the patches are equally frequent.

**
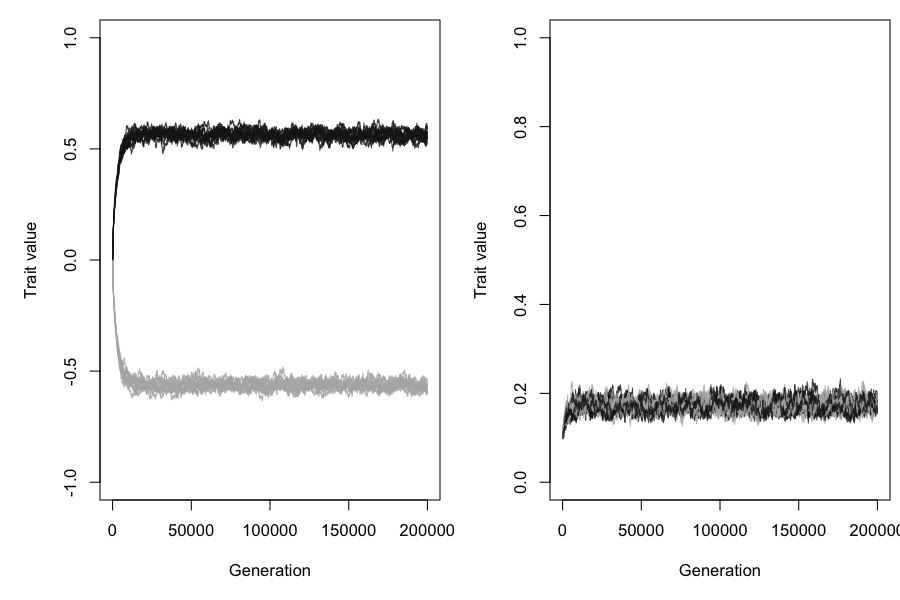
**

**Figure O.** Example trajectories of simulation runs over time for (a) maternal input, *m* (black in crowded environment; grey, in uncrowded environment) and (b) transmission stability, *h* (black for gregarious phenotype, grey for solitary phenotype) for the first combination of simulation parameters in the locust model example 2.1, i.e. when the period of environmental stability *T* is 5, and maternal error *em* is 0.1, and vales of *T* and *em* are symmetric between environments. Note that the starting value for *h* is 0.1.
